# Surface functionalization of coconut-shell-derived activated carbon with amine and surfactant groups for selective short-chain PFAS removal

**DOI:** 10.1039/d6ra07028k

**Published:** 2026-10-05

**Authors:** Yasaman Mohammadi, Hamidreza Sharifan

**Affiliations:** a Department of Chemistry and Biochemistry, Florida Atlantic University Boca Raton FL 33431 USA hsharifan@fau.edu +1-757-287-6370; b School of Environmental, Coastal, and Ocean Sustainability (ECOS), Florida Atlantic University Boca Raton FL 33431 USA; c Department of Chemistry and Biochemistry, University of Texas at El Paso Texas USA

## Abstract

Short-chain per- and polyfluoroalkyl substances (PFAS) pose an increasingly urgent water treatment challenge, as regulatory transitions away from long-chain compounds have accelerated the occurrence of short-chain substitutes that resist adsorption on conventional activated carbon. Here, we engineer the surface chemistry of commercially available coconut-derived activated carbon (CAC), a renewable, highly microporous sorbent (BET surface area: 836.94 m^2^ g^−1^) through amine (ethylenediamine, EDA) and cationic surfactant (cetyltrimethylammonium bromide, CTAB) functionalization to systematically overcome this limitation. Adsorption performance was evaluated for four PFAS of perfluorobutanoic acid (PFBA), perfluorohexanoic acid (PFHxA), perfluorononanoic acid (PFNA), and perfluorooctanesulfonamide (PFOSA) spanning short and long chain lengths across environmentally relevant concentrations (1–16 µg L^−1^), quantified by LC-MS/MS following EPA Method 1633. Pristine CAC achieved >85% removal for long-chain PFAS (PFOSA, PFNA), driven by hydrophobic partitioning and micropore filling, but showed markedly limited affinity for short-chain compounds (PFBA, PFHxA). Surface functionalization fundamentally redirected adsorption mechanisms: EDA–CAC, despite a 20% reduction in BET surface area to 671.02 m^2^ g^−1^ substantially enhanced PFBA and PFNA uptake through protonated amine-mediated electrostatic attraction and hydrogen bonding with anionic headgroups. CTAB–CAC preserved high surface area (822.30 m^2^ g^−1^) while introducing hydrophobic alkyl domains and permanently charged quaternary ammonium sites that synergistically improved PFHxA and PFOSA removal through hydrophobic chain–chain interactions and electrostatic attraction. Across all systems, adsorption generally followed Freundlich behavior more closely than Langmuir behavior (higher *R*^2^) across most, though not all, systems, consistent with energetically heterogeneous, sub-saturation interactions on functionalized carbon surfaces. For pristine CAC, removal efficiency followed the order PFOSA > PFNA > PFHxA > PFBA, reflecting chain-length-dependent hydrophobicity; among the surface-modified sorbents, CTAB–CAC achieved the greatest enhancement for PFOSA and PFHxA, while EDA–CAC achieved the greatest enhancement for PFNA and PFBA, substantially closing this gap for short-chain PFAS. The findings established a rational interfacial engineering of biomass-derived carbons rather than textural optimization alone is a scalable and mechanistically grounded strategy for next-generation PFAS sorbents targeting short-chain contamination in drinking-water systems.

## Abstract 2

Coconut-shell-derived activated carbon was functionalized with amine and surfactant groups to enhance the selective removal of short-chain PFAS from water.

## Graphical abstract

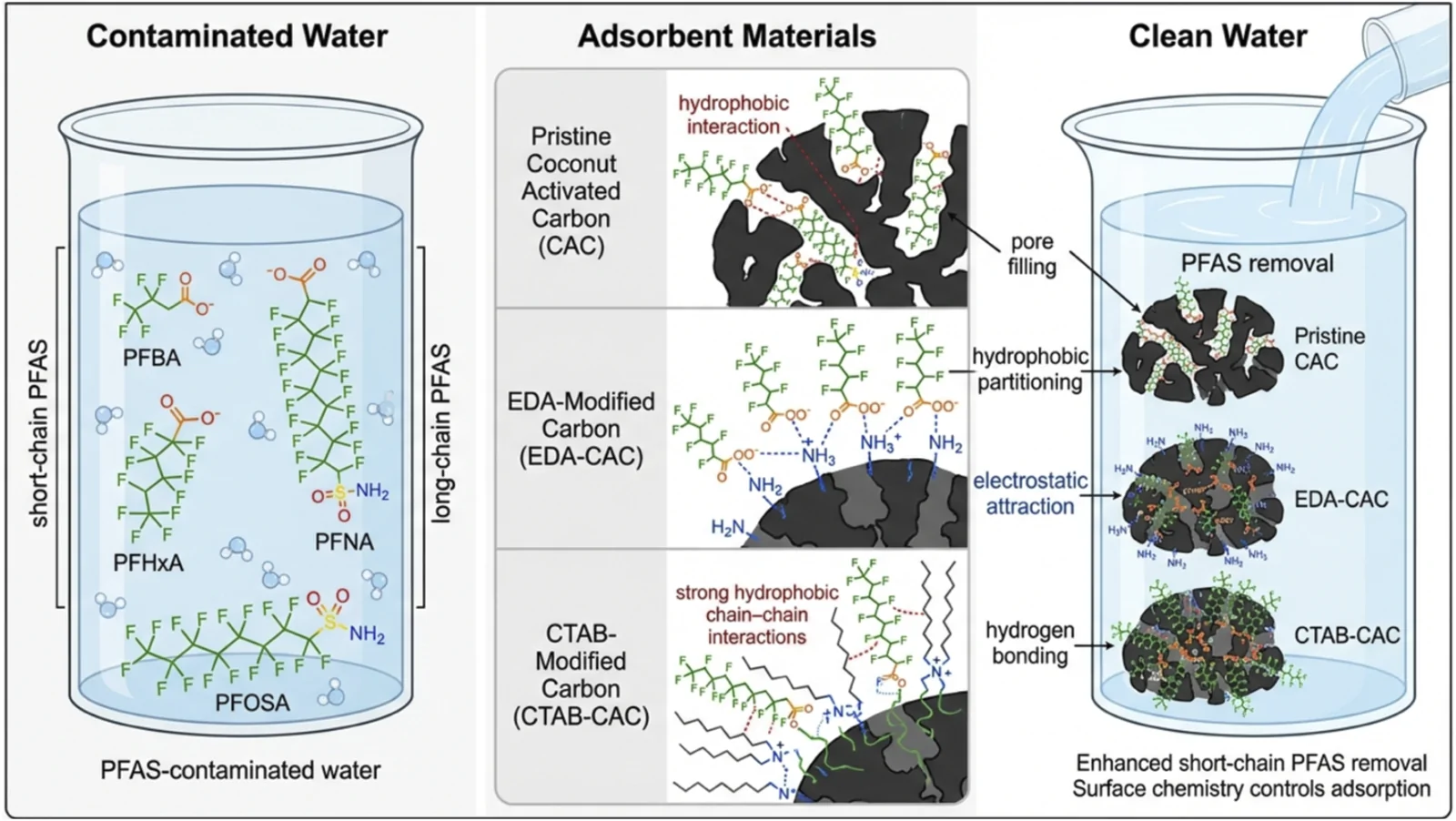

## 1. Introduction

Per- and polyfluoroalkyl substances (PFAS) are a large class of synthetic fluorinated compounds widely used in industrial and consumer products because of their exceptional chemical stability, hydrophobicity, and surfactant properties.^1,2^ These same characteristics have led to their persistence in environmental systems and their global distribution in water, soil, and biological matrices.^3^ PFAS contamination has become a major environmental and public health concern because many compounds in this family exhibit resistance to degradation, long environmental half-lives, and potential toxicological effects including endocrine disruption, immune suppression, and developmental impacts. Consequently, the removal of PFAS from contaminated water has become a critical challenge in environmental engineering and water treatment.^4–6^

Among currently available treatment technologies, adsorption using activated carbon remains one of the most widely implemented and cost-effective approaches for PFAS removal in drinking water and wastewater treatment systems.^7–9^ Granular and powdered activated carbons offer high surface areas, well-developed pore structures, and strong affinity for hydrophobic organic contaminants.^10^ However, adsorption performance strongly depends on the physicochemical properties of PFAS molecules, particularly the length of the perfluorinated carbon chain and the nature of the functional headgroup.^11–13^ Long-chain PFAS typically exhibit strong adsorption on activated carbon due to enhanced hydrophobic interactions and pore-filling mechanisms, whereas short-chain PFAS display significantly weaker affinity because of their lower hydrophobicity and smaller molecular size.^14,15^ This disparity has become an increasing concern as regulatory restrictions on long-chain PFAS have accelerated the use of short-chain substitutes, which are more mobile in aquatic environments and more difficult to remove using conventional adsorbents.^16,17^

For clarity, long-chain PFAS are defined here as perfluoroalkyl carboxylic acids with eight or more carbons (≥C8; *e.g.*, PFOA, C8, MW ≈ 414 g mol^−1^) and perfluoroalkyl sulfonic acids with six or more carbons (≥C6; *e.g.*, PFOS, C8, MW ≈ 500 g mol^−1^), while short-chain PFAS are the corresponding acids below these carbon-number thresholds (*e.g.*, PFBA, C4, MW ≈ 214 g mol^−1^), consistent with widely used OECD/ITRC terminology.^19^

Recent research has therefore focused on improving PFAS adsorption through chemical modification of carbon surfaces to introduce new interaction pathways beyond simple hydrophobic partitioning. Functionalization strategies such as surfactant coating, amine grafting, and surface oxidation can modify surface charge, polarity, and interfacial chemistry, thereby influencing electrostatic interactions, hydrogen bonding, and hydrophobic partitioning with PFAS molecules.^12,13,18^ These modifications may be particularly beneficial for enhancing the adsorption of short-chain PFAS, where intrinsic hydrophobic driving forces are relatively weak.^10,13^ Nevertheless, the relationship between surface functionalization, PFAS molecular structure, and adsorption mechanisms remains insufficiently understood, especially under environmentally relevant concentration ranges where adsorption often occurs below saturation conditions.^19^

Coconut-shell-derived activated carbon represents a particularly attractive sorbent material due to its high microporosity, large surface area, and renewable biomass origin. However, its adsorption behavior toward PFAS is primarily governed by physical interactions associated with its aromatic carbon framework and pore network.^20,21^ Tailoring the surface chemistry of coconut activated carbon therefore offers a promising pathway to enhance PFAS removal by introducing additional interaction mechanisms while maintaining its favorable structural properties.

Prior work has applied cationic-surfactant and amine-based modifications to activated carbon for PFAS removal, though largely for long-chain compounds and rarely as a direct, head-to-head comparison on the same parent carbon. Shaikh *et al.*^34^ demonstrated that CTAB-impregnated activated carbon substantially enhanced PFOA removal through combined electrostatic attraction (*via* the CTAB quaternary ammonium head group) and hydrophobic co-adsorption (*via* its C16 alkyl tail) within a closed-loop, regenerable system. Okorie *et al.*^43^ reviewed a broader set of activated-carbon modification strategies for PFAS treatment, including amine functionalization, cationic-surfactant impregnation, and metal/metal-oxide loading and noted that most reported studies emphasize long-chain PFAS, leaving amine-versus-surfactant comparisons for short-chain congeners comparatively underexplored. More recently, Wang *et al.*^44^ modified granular activated carbon with the closely related quaternary ammonium surfactant cetyltrimethylammonium chloride (CTAC) and reported enhanced adsorption capacities for PFOA (93.18 *versus* 90.53 mg g^−1^ on unmodified GAC) and PFNA (130.94 *versus* 103.71 mg g^−1^), attributed to combined electrostatic and hydrophobic interactions, with the Langmuir model providing the best isotherm fit; notably, competitive co-adsorption in PFOA/PFNA mixtures reduced PFNA removal relative to single-solute conditions, underscoring a key limitation of single-compound testing. The present study extends this body of work by directly comparing amine (EDA) and surfactant (CTAB) functionalization of the same coconut-shell-derived parent carbon, and by targeting short-chain PFAS (PFHxA, PFBA) that remain comparatively understudied relative to long-chain congeners in this literature.

In this study, we investigate the adsorption behavior of four representative PFAS compounds—perfluorobutanoic acid (PFBA), perfluorohexanoic acid (PFHxA), perfluorononanoic acid (PFNA), and perfluorooctanesulfonamide (PFOSA) on pristine coconut activated carbon (CAC) and two chemically modified derivatives. Surface functionalization was performed using ethylenediamine (EDA) to introduce amine groups capable of electrostatic and hydrogen-bond interactions,^22–24^ and cetyltrimethylammonium bromide (CTAB) to generate hydrophobic surfactant domains and positively charged quaternary ammonium groups. These modifications were designed to selectively enhance adsorption pathways for different PFAS structures while preserving the underlying porous carbon framework.

We focused on PFBA, PFHxA, PFNA, and PFOSA to span a deliberate long-to-short perfluoroalkyl chain-length gradient among carboxylic acids (C4–C9) together with a structurally distinct sulfonamide precursor. We acknowledge that perfluoroalkyl sulfonic acids (PFSAs) such as PFOS, PFHxS, and PFBS are generally more toxic and bioaccumulative than the PFCAs studied here and are now individually regulated, together with PFOA, PFNA, and HFPO-DA (GenX), under the U.S. EPA's 2024 PFAS National Primary Drinking Water Regulation, which also establishes a Hazard Index limit for mixtures including PFBS.^45^ PFSAs were outside the scope of the present study; their inclusion, alongside mixed PFCA/PFSA panels under competitive conditions, is identified as a priority for future work (see Conclusion).

The objective of this work is to elucidate how surface chemistry and PFAS molecular structure jointly control adsorption behavior on biomass-derived activated carbon. By systematically comparing pristine and functionalized carbons across PFAS with different chain lengths and functional groups, this study aims to provide mechanistic insight into adsorption processes relevant to drinking-water treatment and environmental remediation. Understanding these structure–surface relationships can guide the rational design of carbon-based sorbents optimized for emerging PFAS contamination scenarios, particularly for the increasingly prevalent short-chain compounds that challenge existing treatment technologies.

## 2. Materials and methods

Comprehensive physicochemical characterization of pristine and modified carbons was performed using scanning electron microscopy (SEM), energy-dispersive X-ray spectroscopy (EDS), Fourier transform infrared spectroscopy (FTIR), BET surface area analysis, and zeta potential measurements. Batch adsorption experiments were conducted at environmentally relevant concentrations (1–16 µg L^−1^), and adsorption equilibria were analyzed using Langmuir and Freundlich isotherm models to evaluate adsorption capacity and surface heterogeneity. PFAS concentrations were quantified by LC-MS/MS following EPA Method 1633 to ensure high analytical sensitivity and accuracy.

### 2.1. Materials

Coconut-shell-derived activated carbon (CAC) was purchased from Viva Doria Inc. and used as the base sorbent material for all modification and adsorption experiments. Ethylenediamine (EDA, ≥99%), cetyltrimethylammonium bromide (CTAB, ≥99%), sodium hydroxide (NaOH, ≥98%), hydrochloric acid (HCl, 37%), sodium chloride (NaCl), anhydrous ethanol (≥99.5%), and all per- and polyfluoroalkyl substances (PFAS) standards, perfluorobutanoic acid (PFBA), perfluorohexanoic acid (PFHxA), perfluorononanoic acid (PFNA), and perfluorooctanesulfonamide (PFOSA) were purchased from Sigma-Aldrich (St. Louis, MO, USA). The names, abbreviations, and chemical structures of the PFAS investigated in this study are provided in the SI, Table S1.

### 2.2. Characterization of sorbents

#### 2.2.1. Surface morphology and elemental composition (SEM-EDS)

The morphology of pristine and modified coconut activated carbon samples was examined using scanning electron microscopy (SEM) with a JEOL JSM-6360 microscope operated at an accelerating voltage of 15 kV. Prior to analysis, dried carbon powders were mounted on silicon wafer chips. Elemental composition was determined using energy-dispersive X-ray spectroscopy (EDS) attached to the SEM system. Spectra were collected from representative regions of each sample to confirm the major and minor elemental components present in the carbon matrix.^21,25^

#### 2.2.2. Surface functional group analysis (FTIR)

Fourier transform infrared (FTIR) spectroscopy was used to identify surface functional groups and evaluate chemical changes after EDA and CTAB modification.^18,26^ Spectra were collected following ASTM E1252 using a Thermo Scientific Nicolet Apex FTIR spectrometer (Waltham, MA, USA). Dried activated carbon powders were analyzed directly as finely ground solids. FTIR spectra were recorded over the range of 4000–400 cm^−1^ under identical acquisition conditions for all samples. All spectra were obtained in triplicate (*n* = 3) with identical patterns.

#### 2.2.3. Textural properties (BET surface area)

Specific surface area and pore characteristics were determined using multipoint Brunauer–Emmett–Teller (BET) nitrogen adsorption with a HORIBA SA-9600 surface area analyzer.^27^ Prior to analysis, samples were degassed at 300 °C for 120 min to remove adsorbed moisture and volatile impurities. The degassed sample cell was immersed in liquid nitrogen, and nitrogen adsorption–desorption measurements were performed. Three adsorption–desorption cycles were recorded for each sample. The BET surface area was calculated using instrument software and normalized by the degassed sample mass.

### 2.3. Surface charge and point of zero charge (zeta potential)

The electrokinetic surface charge of pristine and modified coconut activated carbon samples was determined using a Malvern Zetasizer (Malvern Panalytical, UK) based on electrophoretic light scattering. Carbon suspensions were prepared by dispersing the sorbent at a concentration of 0.05 g L^−1^ in 0.01 M NaCl solution, which served as a background electrolyte to maintain constant ionic strength during measurements. The pH of the suspensions was adjusted over the range of 2–12 using dilute HCl or NaOH solutions. After pH adjustment, suspensions were gently mixed and allowed to equilibrate prior to measurement to ensure stabilization of the surface charge. Zeta potential values were measured as a function of pH to determine the electrokinetic behavior of the carbon surfaces and estimate the point of zero charge (PZC). These results were subsequently used to guide the selection of solution pH for adsorption experiments to control surface charge conditions and PFAS speciation. All measurements were performed in triplicate (*n* = 3), and reported values represent the mean of independent measurements.^26,28^

### 2.4. Preparation of ethylenediamine-modified coconut activated carbon (EDA–CAC)

EDA-functionalized coconut activated carbon was prepared by a wet-impregnation method.^18^ Briefly, a 20% (v/v) ethylenediamine (EDA) solution was prepared in anhydrous ethanol. Because the solution was strongly basic (pH > 11) due to the excess amine, it was partially neutralized with diluted HCl to pH 9 to moderate the reaction environment while maintaining amine availability for surface functionalization. An ethanol/water mixture (1 : 1, v/v) was then used as the impregnation medium. Coconut activated carbon powder was added to the solution at a solid-to-liquid ratio of 1 : 10 (w/v), and the suspension was continuously stirred at 80 °C for 6 h to promote diffusion of EDA into the pore structure and interaction with surface oxygen-containing functional groups. These conditions (temperature, contact time, and solid-to-liquid ratio) were selected based on preliminary optimization trials showing diminishing functionalization gains beyond 6 h and thermal degradation risk above 80 °C. After modification, the carbon was recovered and washed repeatedly with deionized water until the rinse water reached neutral pH, indicating removal of unreacted or weakly bound EDA. The washed material was then dried in an oven at 105 °C for 24 h and stored in a sealed container until further characterization and adsorption experiments.

### 2.5. Preparation of CTAB-functionalized coconut activated carbon (CTAB–CAC)

CTAB functionalization of coconut activated carbon was carried out through a two-step modification procedure involving alkaline pretreatment followed by surfactant impregnation.^12^ Initially, pristine CAC was subjected to alkaline washing to remove soluble impurities and enhance the availability of surface functional groups. For this step, CAC was dispersed in 2 M NaOH solution at a solid-to-liquid ratio of 1 : 10 (w/v) and stirred at 80 °C for 4 h. The alkaline pretreatment temperature/duration and the subsequent CTAB loading conditions (50 °C, 18 h, 1 mM CTAB) were likewise selected. The treated carbon was subsequently washed several times with deionized water and then with diluted HCl until the suspension reached approximately neutral pH (≈7). The pretreated material was dried in an oven at 105 °C for 24 h. In the second step, the pretreated CAC was functionalized with cetyltrimethylammonium bromide (CTAB). The dried carbon was dispersed in an aqueous CTAB solution (1 mM) at a ratio of 1 : 10 (w/v) and stirred at 50 °C for 18 h to allow adsorption of the surfactant onto the carbon surface and within accessible pore structures. Following functionalization, the carbon was recovered and washed repeatedly with deionized water until the pH and conductivity of the rinse water stabilized (≈6) and no visible foaming was observed, indicating the removal of unbound CTAB molecules. The resulting CTAB-modified carbon was then dried at 105 °C for 24 h and stored in sealed containers prior to characterization and adsorption experiments.

### 2.6. Batch adsorption experiments

Batch adsorption experiments were conducted to evaluate the removal of PFAS by pristine coconut activated carbon (CAC) and surface-modified carbons (EDA–CAC and CTAB–CAC).^29,30^ Stock solutions of perfluorobutanoic acid (PFBA), perfluorohexanoic acid (PFHxA), perfluorononanoic acid (PFNA), and perfluorooctanesulfonamide (PFOSA) were prepared in 18.2 MΩ cm deionized water. Working solutions were prepared at initial concentrations (*C*_0_) of 1, 2, 4, 8, and 16 µg L^−1^. This concentration range was selected to reflect reported water pollution to PFAS,^1^ and spanning from near-environmentally-relevant to higher bench-scale concentrations needed to generate full adsorption isotherms. Batch adsorption tests were conducted using 50 mL PFAS solutions with a sorbent dosage of 50 mg L^−1^, corresponding to 2.5 mg of sorbent per 50 mL tube. Pristine CAC was evaluated for adsorption of all four PFAS compounds to provide a baseline comparison. The modified sorbents were tested selectively according to their targeted adsorption mechanisms: EDA–CAC was evaluated for PFBA and PFNA, while CTAB–CAC was tested for PFHxA and PFOSA. This pairing was hypothesis-driven as EDA's primary amine groups were expected to favor electrostatic/hydrogen-bonding interactions most beneficial to the more polar, shorter-chain carboxylates (PFBA, PFNA), whereas CTAB's long hydrophobic tail and quaternary ammonium head were expected to favor the more amphiphilic/hydrophobic compounds (PFOSA, PFHxA). We acknowledge that this design, while hypothesis-driven and resource-efficient, precludes a full cross-comparison; testing the remaining four sorbent–PFAS combinations (EDA–CAC *vs.* PFOSA/PFHxA and CTAB–CAC *vs.* PFBA/PFNA) is identified as a priority for follow-up work. The solution pH for all experiments was adjusted to 5.5 based on zeta potential characterization of the sorbents to ensure environmentally relevant PFAS speciation and favorable surface charge conditions. Reaction tubes were sealed and agitated on an orbital shaker at 150 rpm at room temperature (22–25 °C) for 12 h, a contact time commonly used in PFAS adsorption studies to ensure equilibrium conditions. All experiments were conducted in triplicate (*n* = 3). To account for potential PFAS losses due to container adsorption or handling, no-sorbent control blanks consisting of PFAS solutions in identical polypropylene tubes were included in parallel. After equilibration, suspensions were centrifuged at 5000 rpm for 10 min. The resulting supernatants were filtered through 0.22 µm polypropylene syringe filters into polypropylene LC-MS/MS vials and stored at 4 °C prior to analysis. Regarding possible micelle or aggregate formation during filtration, reported critical micelle concentrations (or aggregation onsets, which for several short-chain PFAS are not classical micellization) for perfluoroalkyl acids generally increase as chain length decreases, with literature values on the order of several to tens of millimolar for long-chain congeners and even higher, or not clearly defined, for short-chain congeners such as PFHxA and PFBA. Our experimental initial concentrations (1–16 µg L^−1^) are several orders of magnitude below these millimolar-scale thresholds, so we do not expect micelle/aggregate formation or retention on the 0.22 µm filters to have measurably affected the reported results; this is stated here as a reasoned assumption based on concentration scaling rather than an experimentally verified absence of aggregation. The equilibrium adsorption capacity (*q*_e_, µg g^−1^) was calculated according to eqn (1):1
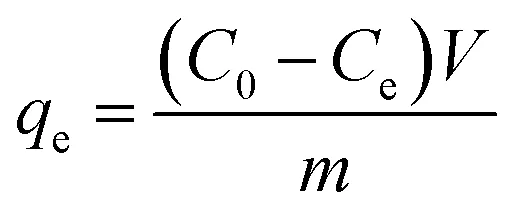
where *C*_0_ and *C*_e_ are the initial and equilibrium PFAS concentrations (µg L^−1^), *V* is the solution volume (L), and *m* is the mass of CAC (g). Removal efficiency in percentage (%) was calculated using this equation, as shown in eqn (2):2
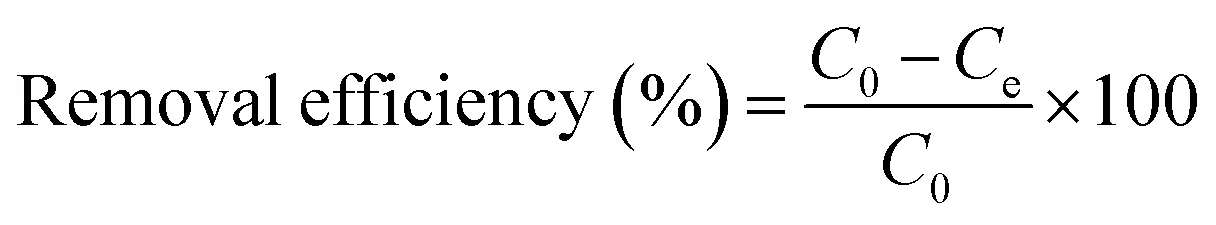
where *C*_0_ is the initial PFAS concentration and *C*_e_ is the equilibrium (final) concentration after adsorption (both in µg L^−1^).

### 2.7. LC-MS/MS analysis

PFAS concentrations in aqueous samples were quantified using liquid chromatography-tandem mass spectrometry (LC-MS/MS) with a Shimadzu LCMS-8050 triple quadrupole system operated according to EPA Method 1633.^31^ The instrument was operated in negative electrospray ionization mode (ESI^−^) using multiple reaction monitoring (MRM) for selective detection of target analytes. Chromatographic separation and mass spectrometric parameters were implemented following the conditions specified in EPA Method 1633. Calibration standards were prepared in 18.2 MΩ cm deionized water over a concentration range of 0.01–20 µg L^−1^. Calibration curves were generated using weighted linear regression (1/*x* weighting) to account for heteroscedasticity at low concentrations. Samples exceeding the calibration range were diluted with deionized water prior to analysis to ensure accurate quantification. Following batch adsorption experiments, supernatants were collected after centrifugation and filtered through 0.22 µm polypropylene syringe filters into polypropylene LC-MS/MS vials to minimize PFAS adsorption to container surfaces. Filtered samples were stored at 4 °C and analyzed within the recommended holding time to prevent potential analyte degradation. Quality assurance and quality control (QA/QC) procedures were implemented in accordance with EPA Method 1633. These included method blanks and solvent blanks to assess background contamination, matrix blanks and matrix spike samples to evaluate recovery and matrix effects, and duplicate samples to assess analytical precision. Continuing calibration verification (CCV) standards were analyzed at the beginning of each analytical batch and after every ten injections, with acceptance criteria of ±20% of the expected value. Matrix spike recoveries were considered acceptable within the range of 70–130%, and duplicate precision was evaluated using relative percent difference (RPD ≤ 20%). Method detection limits (MDLs) and limits of quantification (LOQs) were determined following the procedures outlined in EPA Method 1633. The calibration curves of the analyzed PFAS are provided in SI (Fig. S3). EPA Method 1633 was selected because it is the U.S. EPA's multi-laboratory validated, isotope-dilution LC-MS/MS method for PFAS in aqueous matrices and is increasingly required by state and federal regulators for defensible PFAS data; its extraction, cleanup, and QA/QC framework was applied here even though only four analytes were the focus of this study.

### 2.8. Adsorption isotherm modeling

Adsorption equilibrium data were analyzed using the nonlinear forms of the Langmuir and Freundlich isotherm models.^32^ Nonlinear regression was applied directly to the original model equations to avoid potential bias and error propagation associated with linearized transformations.

The Langmuir isotherm model assumes monolayer adsorption onto a homogeneous surface with a finite number of identical adsorption sites and is expressed as:3
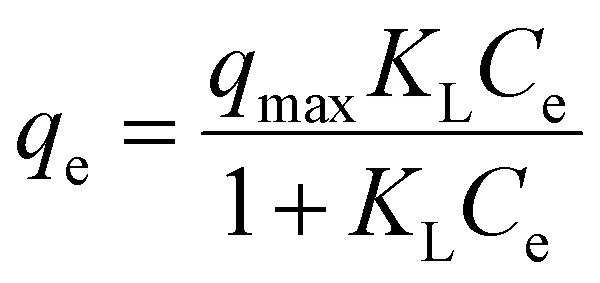
where *q*_e_ (µg g^−1^) is the equilibrium adsorption capacity, *q*_max_ (µg g^−1^) is the maximum monolayer adsorption capacity, *K*_L_ (L µg^−1^) is the Langmuir affinity constant, and *C*_e_ (µg L^−1^) is the equilibrium solute concentration. The Freundlich isotherm model, which describes adsorption on heterogeneous surfaces with a nonuniform distribution of adsorption energies, is given by:

4 *q*_e_ = *K*_F_*C*_e_^1/*n*^

where *K*_F_ [(µg g^−1^) (L µg^−1^)^1/*n*^] is the Freundlich adsorption constant related to adsorption capacity, and *n* is the heterogeneity factor, with *n* > 1 indicating favorable adsorption. Model parameters were obtained using nonlinear least-squares regression. The goodness of fit for each model was evaluated using the coefficient of determination (*R*^2^), calculated based on the agreement between experimental and model-predicted *q*_e_ values.

## 3. Results and discussion

### 3.1. Characterization of CAC

#### 3.1.1. Scanning electron microscopy and energy-dispersive X-ray spectroscopy analysis

Scanning electron microscopy (SEM) image of pristine coconut activated carbon that is shown in Fig. 1A, shows a highly irregular, porous, and fragmented surface morphology. The activated carbon displays interconnected voids, cavity-like features, and rough carbon textures at the micro- to macro-scale, characteristic of biomass-derived chars produced by pyrolysis.^21,25^ The heterogeneous surface texture and visible pore network suggest abundant accessible sites and a broad distribution of adsorption domains.^21,25^ No distinct crystalline phases or smooth surfaces are observed, supporting the amorphous carbonaceous nature of the material and its suitability as a sorbent for organic contaminants. Energy-dispersive X-ray spectroscopy (EDS) analysis of pristine CAC confirms that the material is predominantly carbon-based, with carbon and oxygen as the major constituents, Fig. 1B. The elemental composition shows a high carbon content, 83.5 at%, accompanied by oxygen, 12.8%, indicating the presence of oxygen-containing surface functional groups such as hydroxyl, carbonyl, and carboxyl moieties.^33^

**Fig. 1:**
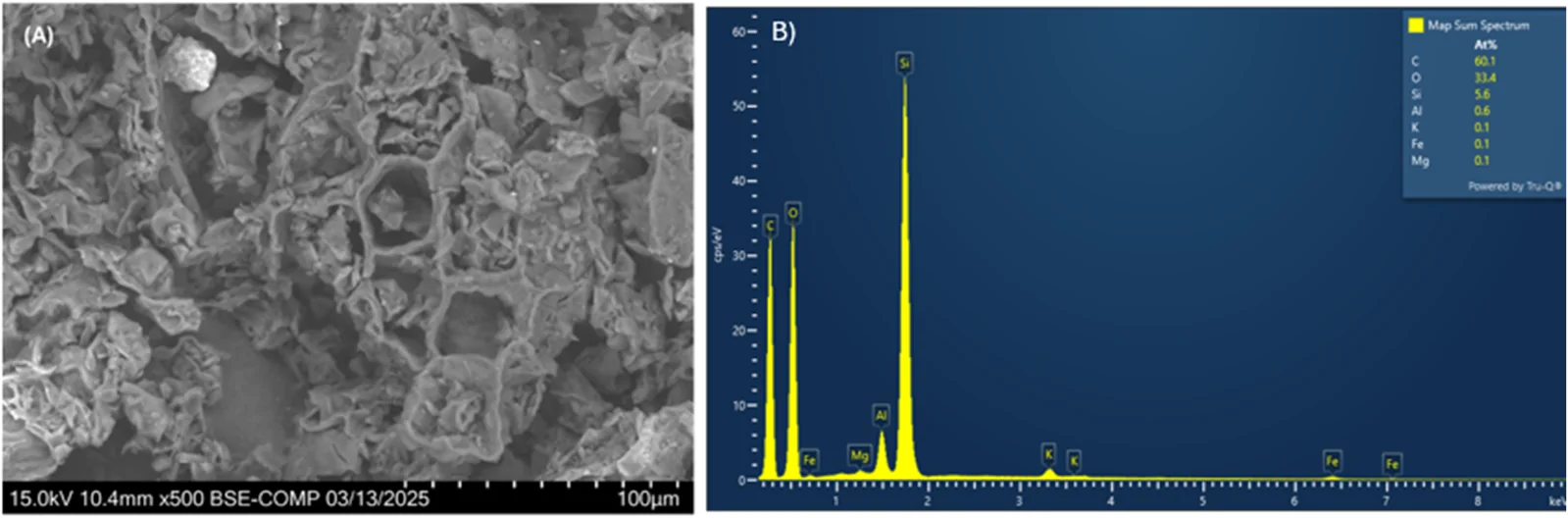
(A) Scanning electron microscopy (SEM) image of coconut activated carbon (CAC) showing the porous surface morphology; (B) energy-dispersive X-ray spectroscopy (EDS) spectrum confirming elemental composition of the CAC matrix.

#### 3.1.2. FTIR analysis

Fig. 2A presents the FTIR spectra of pristine CAC, EDA–CAC, CTAB–CAC, highlighting surface chemical changes after modification. The observed peaks are characteristic of highly aromatic carbon materials. Pristine CAC exhibits bands at around 1700 cm^−1^ (C=O stretching), 1580 cm^−1^ (aromatic C=C), and 1000–1100 cm^−1^ (C–O stretching), indicating the presence of oxygen-containing functional groups and aromatic carbon structures typical of activated carbon.^26^ Following amine functionalization, new bands appear at 1550–1650 cm^−1^ (N–H bending) and 1000–1100 cm^−1^ (C–N stretching), confirming successful incorporation of nitrogen-containing groups. Additional lower-frequency bands observed for EDA–CAC in the 750–1000 cm^−1^ region are attributed to out-of-plane N–H wagging vibrations of the grafted primary amine (–NH_2_) groups a well-established complementary band to the N–H bending (1550–1650 cm^−1^) and C–N stretching (1000–1050 cm^−1^) signatures already discussed above providing further FTIR-based confirmation, further supporting successful amine functionalization.^18,34^ These functionalities introduce positively charged sites under acidic to neutral conditions, promoting electrostatic interactions with anionic PFBA, PFHxA, PFNA and explaining enhanced low-concentration adsorption despite reduced BET surface area. CTAB–CAC exhibits distinct –CH_2_ stretching bands at around 2920 and 2850 cm^−1^, characteristic of long alkyl chains, confirming effective surfactant coating. This is consistent with prior CTAB/CTAC-modified activated/granular activated carbon studies for PFOA and PFNA, which likewise attribute enhanced removal to combined electrostatic (quaternary ammonium) and hydrophobic (alkyl chain) interactions.^12,34,44^ The combined presence of hydrophobic alkyl domains and positively charged quaternary ammonium groups likely enhances PFAS adsorption through hydrophobic partitioning and electrostatic attraction.^13,28^

**Fig. 2:**
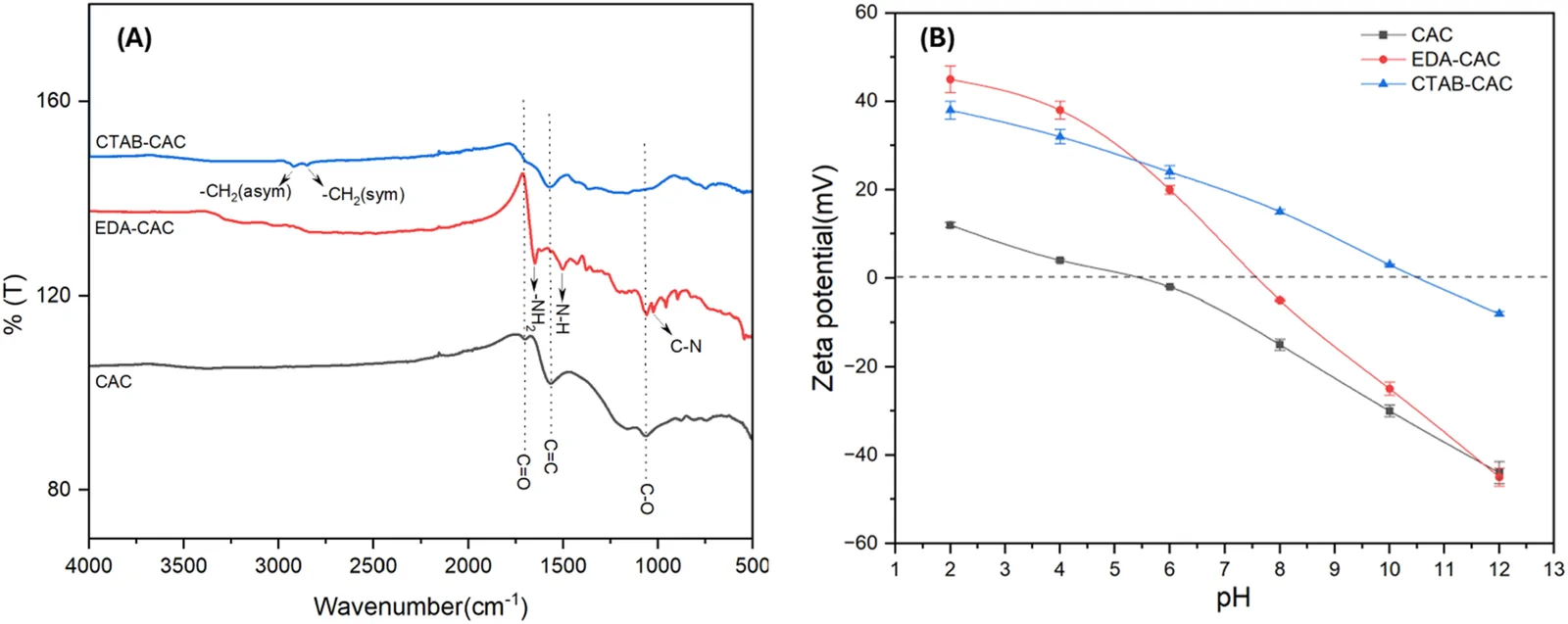
(A) FTIR spectra of CAC, EDA–CAC, and CTAB–CAC, illustrating surface functional-group modifications induced by chemical treatments. (B) Zeta potential of CAC, EDA–CAC, and CTAB–CAC as a function of pH.

#### 3.1.3. BET surface area analysis

The textural properties of pristine and modified CAC were characterized using N_2_ adsorption–desorption BET analysis, and the results are summarized in Table 1. Pristine CAC exhibited a specific surface area of 836.94 m^2^ g^−1^ with a BET C constant of 100.14, indicating a predominantly microporous structure with strong adsorbate–surface interactions. Generating full isotherm plots (and *t*-plot/BJH pore-size distributions) would require re-analysis on an instrument capable of exporting raw isotherm data, which lead to a direction for more detailed textural characterization in future work. EDA–CAC led to a substantial reduction in BET surface area to 671.02 m^2^ g^−1^ and a slightly lower BET *C* constant (96.85), reflecting partial pore blockage, likely associated at least in part with incorporation of amine functional groups near or within the micropore network, resulting in a mixed micro–mesoporous structure.^25^ Despite the reduced surface area, the introduction of nitrogen-containing functionalities is expected to enhance specific interactions with anionic contaminants.

**Table 1:** BET surface area, BET *C* constant, and dominant pore characteristics of pristine coconut activated carbon (CAC), amine-functionalized CAC (EDA–CAC), and CTAB-functionalized CAC (CTAB–CAC)

Material	BET surface area (m^2^ g^−1^)	BET *C* constant	Dominant pore type
CAC	836.94	100.14	Microporous
EDA–CAC	671.02	96.85	Micro–mesoporous
CTAB–CAC	822.30	122.29	Strongly microporous

In contrast, CTAB–CAC retained a high BET surface area (822.3 m^2^ g^−1^) comparable to pristine CAC, along with an increased BET C constant of 122.29. This suggests preservation of the microporous framework and enhanced surface adsorption energy, likely due to surfactant coating along pore walls rather than extensive pore blocking.^21^ These results demonstrate that surface functionalization substantially alters adsorption chemistry while affecting porosity to varying extents depending on the modification strategy.^3^

### 3.2. pH and zeta potential measurements of pristine and modified CAC

Fig. 2B presents the zeta potential of pristine CAC, EDA–CAC, CTAB–CAC as a function of pH. Pristine CAC exhibits a gradual transition from weakly positive to negative surface charge with increasing pH, with a point of zero charge (PZC) near pH 5.5, consistent with deprotonation of oxygen-containing surface groups. EDA–CAC shows strongly positive zeta potential under acidic to near-neutral conditions due to protonated amine functionalities, followed by a decrease in surface charge as pH increases and amine groups deprotonate. CTAB–CAC maintains a positive surface charge across a broader pH range because of permanently charged quaternary ammonium groups.

Adsorption experiments were conducted at pH 5.5 for CAC, EDA–CAC, and CTAB–CAC. This pH was selected to maintain carboxylate PFAS (PFBA, PFHxA, and PFNA) predominantly in their anionic form under environmentally relevant conditions and as well as ensure favorable sorbent surface charge characteristics. At pH 5.5, pristine CAC is near its PZC, minimizing electrostatic repulsion and allowing non-electrostatic interactions, such as hydrophobic partitioning, to contribute significantly.^26^ Both modified carbons remain positively charged at this pH, promoting electrostatic attraction with anionic PFBA, PFHxA, and PFNA and enhancing adsorption through charge-assisted mechanisms.^35,36^ in contrast, PFOSA, a sulfonamide PFAS, is generally considered neutral over a wide pH range; thus, its adsorption is expected to be governed primarily by hydrophobic interactions rather than electrostatic forces.^37^ This controlled pH approach ensures that observed adsorption trends mainly reflect differences in surface chemistry and interfacial interactions rather than variations in PFAS speciation.

### 3.3. Adsorption behavior and surface modification effects of long chains

Fig. 3A and C present the adsorption equilibrium behavior of PFOSA and PFNA, respectively, on pristine CAC, while panels Fig. 3B and D present the corresponding behavior on the surface-functionalized carbons (CTAB–CAC and EDA–CAC, respectively). Experimental equilibrium data were fitted using the nonlinear forms of the Langmuir and Freundlich isotherm models to evaluate adsorption capacity and surface heterogeneity. Overall, the Freundlich model provided better statistical agreement with the experimental data than the Langmuir model for most, but not all, of the adsorption systems examined. Rather than indicating a single, universal adsorption mechanism, this pattern suggests that PFAS adsorption most often occurs *via* multiple, potentially concurrent interaction mechanisms on energetically heterogeneous surfaces, rather than through ideal monolayer adsorption on uniform sites alone. Such behavior is expected for biomass-derived activated carbons, which typically exhibit a wide distribution of pore sizes and surface functional groups resulting from their pyrolytic formation and subsequent surface modification.

**Fig. 3:**
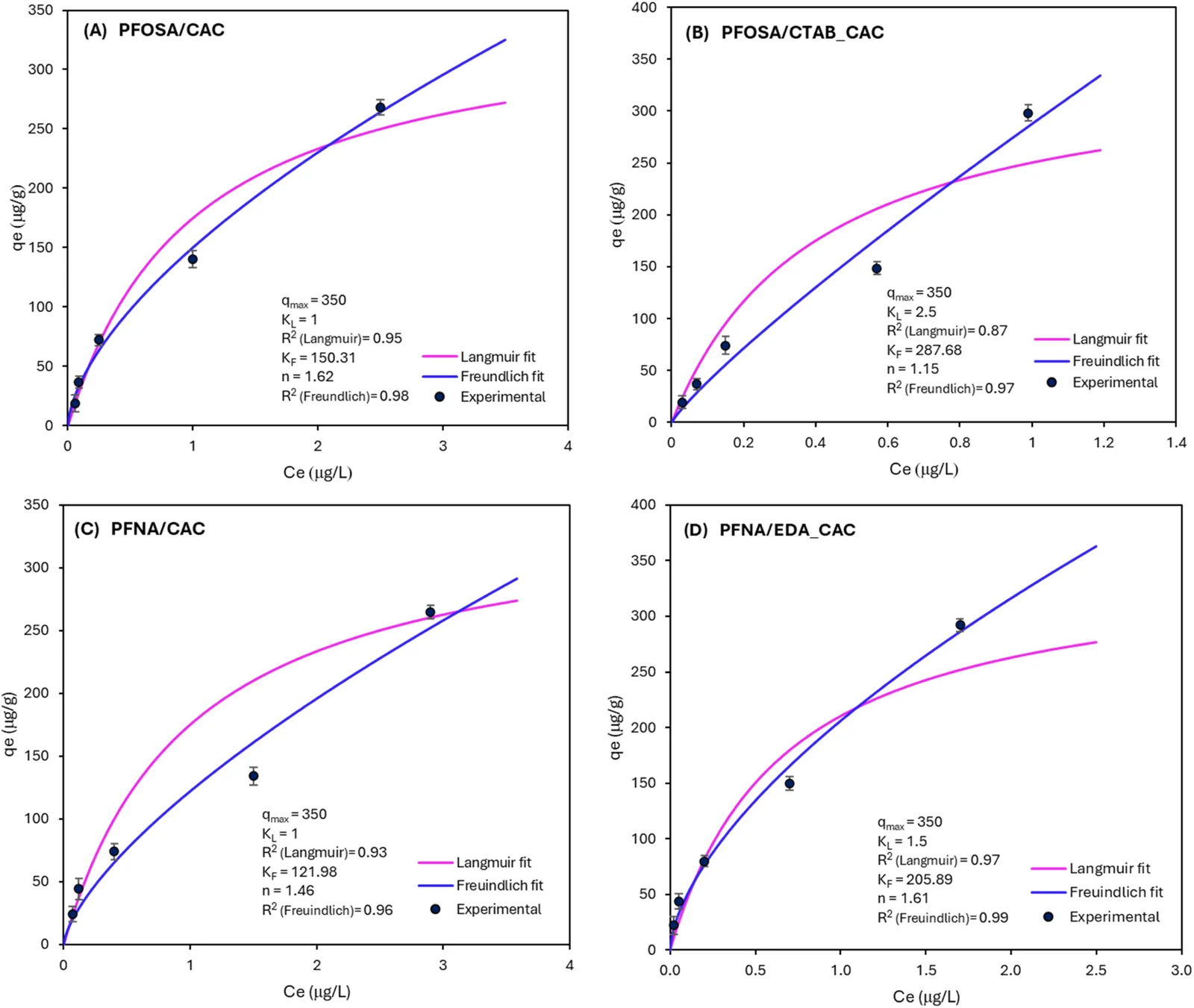
Langmuir and Freundlich isotherm model fits for PFAS adsorption on pristine coconut activated carbon (CAC) and surface-functionalized CAC. (A) PFOSA on CAC, (B) PFOSA on CTAB–CAC, (C) PFNA on CAC, and (D) PFNA on EDA–CAC. The same figure with same *x* and *y* axis is provided in SI, Fig. S3 for sake of comparison.

For the long-chain PFAS compound PFOSA, both pristine CAC and CTAB-functionalized CAC exhibited strong adsorption across the studied concentration range. The adsorption curves show rapid increases in adsorption capacity at low equilibrium concentrations followed by gradual curvature, indicating high affinity adsorption under sub-saturation conditions. This behavior is characteristic of hydrophobic organic compounds interacting with microporous carbon surfaces.^7,38^ The Freundlich model provided an excellent fit to the PFOSA adsorption data for both sorbents, indicating heterogeneous adsorption dominated by multiple interaction mechanisms. Long-chain PFAS such as PFOSA possess extended perfluorinated carbon chains that promote strong hydrophobic interactions with the aromatic carbon framework of activated carbon materials.^39^ These interactions are further enhanced by pore-filling effects within the microporous structure of coconut-derived carbon.

CTAB modification resulted in a noticeable increase in adsorption affinity compared with pristine CAC, despite only minor differences in BET surface area between the two materials. AB modification resulted in a noticeable increase in adsorption affinity compared with pristine CAC, approximately a 91% increase in equilibrium adsorption capacity at *C*_e_ = 1 µg L^−1^ (150.3 *vs.* 287.7 µg g^−1^, based on the Freundlich fits in Fig. 3A and B) despite only minor differences in BET surface area between the two materials. The improved adsorption performance can be attributed to the introduction of hydrophobic alkyl chains and positively charged quaternary ammonium groups on the carbon surface. These surfactant-derived domains can promote PFAS adsorption through enhanced hydrophobic partitioning between the perfluoroalkyl chains of PFOSA and the long hydrocarbon chain of CTAB, while electrostatic attraction between the sulfonamide headgroup and positively charged ammonium sites may also contribute to adsorption.^30^ The combination of hydrophobic and electrostatic interactions likely explains the higher adsorption affinity observed for CTAB–CAC. Although the Langmuir model produced reasonable fits for PFOSA adsorption, the absence of a clear saturation plateau within the studied concentration range indicates that true monolayer coverage was not reached under the experimental conditions. Consequently, the Langmuir-derived maximum adsorption capacities should be interpreted as extrapolated values rather than experimentally confirmed limits.

Similar adsorption behavior was observed for PFNA, although the impact of surface modification differed compared with PFOSA. PFNA adsorption on pristine CAC displayed strong uptake across the tested concentration range, reflecting the significant hydrophobic character of this long-chain perfluorocarboxylic acid. As with PFOSA, the Freundlich model provided the best description of the adsorption data, indicating heterogeneous adsorption behavior on the carbon surface.^38^ Functionalization with ethylenediamine significantly enhanced PFNA adsorption affinity despite a reduction in BET surface area following amine modification. This observation highlights the dominant role of surface chemistry in controlling PFAS adsorption. The introduction of amine functional groups is likely to increase PFNA adsorption through several complementary mechanisms. Under moderately acidic to near-neutral conditions, protonated amine groups can generate positively charged surface sites that promote electrostatic attraction with the anionic carboxylate headgroup of PFNA.^22,40^ In addition, hydrogen bonding interactions between PFNA and surface amine groups may further stabilize adsorption complexes.^18,24^ The enhanced adsorption observed for EDA–CAC demonstrates that chemical functionalization can compensate for reductions in physical surface area by introducing stronger specific interactions between the sorbent surface and PFAS molecules. This finding is consistent with previous studies showing that nitrogen-functionalized carbon materials often display improved adsorption performance for anionic contaminants due to the presence of protonated amine sites.^18,23^

Across all adsorption systems, adsorption strength followed the expected trend associated with PFAS molecular structure and hydrophobicity. Long-chain PFAS such as PFOSA and PFNA exhibit stronger adsorption due to increased hydrophobic interactions and greater van der Waals forces between the fluorinated carbon chain and the carbon surface.^27,41^ In contrast, shorter-chain PFAS generally exhibit weaker adsorption because their reduced hydrophobicity limits interaction with carbonaceous sorbents.^17^ The results further demonstrate that adsorption performance is not controlled solely by surface area or pore volume. Instead, surface chemistry plays a critical role in governing PFAS adsorption behavior. Functionalization with CTAB enhances hydrophobic partitioning and electrostatic interactions, while EDA modification introduces amine groups capable of electrostatic attraction and hydrogen bonding. These modifications alter the interfacial chemistry of the carbon surface, thereby influencing the dominant adsorption mechanisms. The observed adsorption behavior highlights the importance of tailoring sorbent surface chemistry to the molecular characteristics of target PFAS contaminants. Pristine activated carbon remains highly effective for long-chain PFAS due to strong hydrophobic interactions within its microporous structure. However, surface-functionalized carbons may provide additional advantages for improving adsorption affinity and expanding treatment capabilities, particularly under low-concentration conditions typical of drinking-water contamination (often in the low-ng L^−1^ to low-µg L^−1^ range).^45^

### 3.4. Adsorption behavior and surface modification effects of short-chain PFAS

Fig. 4A–D shows the adsorption behavior of PFHxA and PFBA on pristine CAC and modified carbons (CTAB–CAC and EDA–CAC). As with long-chain PFAS, the Freundlich model generally provided slightly better agreement with the experimental data, indicating adsorption on heterogeneous surfaces. However, the adsorption behavior of short-chain PFAS differed markedly, with significantly lower adsorption capacities and a stronger dependence on surface functionalization. This reflects the reduced hydrophobicity and smaller molecular size of short-chain compounds, which limit both hydrophobic partitioning and pore-filling contributions. The adsorption experiments were run in triplicate (*n* = 3; see Section 2.5) and that error bars in Fig. 4 represent ± SD of triplicate measurements.^17,27^

**Fig. 4:**
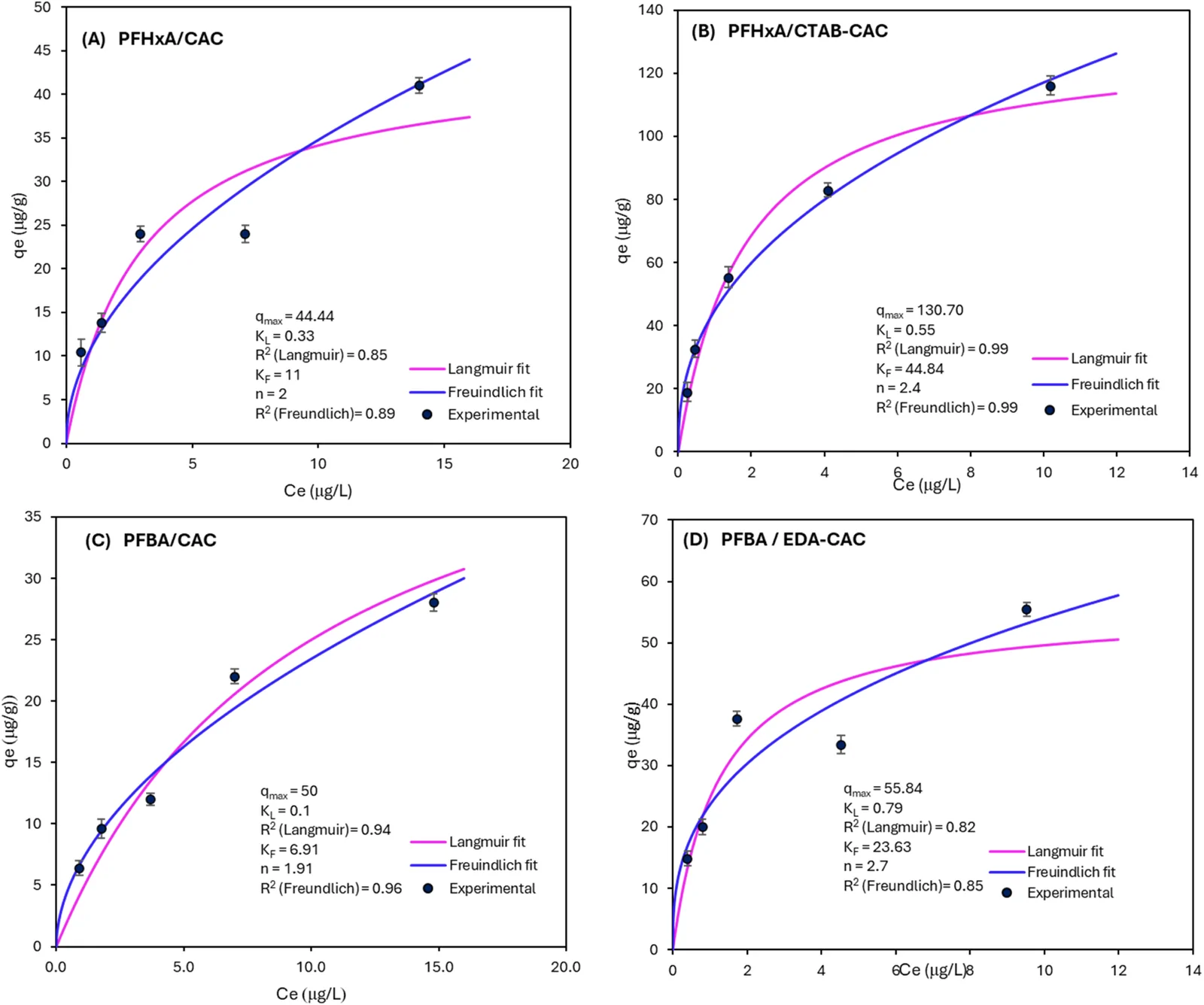
Langmuir and the Freundlich isotherm models fit for PFAS adsorption on pristine coconut activated carbon (CAC) and surface-functionalized CAC. Panels compare paired sorbents for each PFAS: (A) PFHxA on CAC, (B) PFHxA on CTAB–CAC, (C) PFBA on CAC, and (D) PFBA on EDA–CAC. The same figure with same *x* and *y* axis is provided in SI, Fig. S4 for sake of comparison.

For PFHxA, adsorption on pristine CAC was relatively weak overall; equilibrium uptake (*q*_e_) initially decreased over the lower range of equilibrium concentrations (*C*_e_) before increasing again at higher *C*_e_ (Fig. 4A), a non-monotonic pattern that may reflect initial surface-site saturation/blocking effects followed by a secondary uptake mechanism (*e.g.*, hydrophobic partitioning or surface aggregation) at higher *C*_e_, rather than a single, low-affinity trend. Both Langmuir and Freundlich models provided moderate fits, with Freundlich slightly outperforming Langmuir, consistent with adsorption on heterogeneous surface sites.^7,37^ The reduced adsorption can be attributed to the shorter perfluorinated chain, which weakens hydrophobic interactions with the carbon surface and reduces the driving force for adsorption.^28^ As a result, PFHxA uptake on pristine CAC appears to be governed primarily by relatively weak interfacial interactions rather than strong pore-filling effects.^25^

In contrast, CTAB-functionalized CAC exhibited substantially improved PFHxA adsorption across the tested concentration range (Fig. 4B). Removal efficiencies increased significantly, and the adsorption isotherm became more well-defined, with both models showing excellent agreement. The marked increase in adsorption capacity despite minimal changes in surface area highlights the importance of surface chemistry in controlling adsorption behavior.^12^ The hydrophobic domains introduced by CTAB likely enhance partitioning of PFHxA, while the positively charged ammonium groups may contribute additional electrostatic interactions.^34^ Together, these effects reduce the energetic barrier associated with adsorption of short-chain PFAS and promote stronger interaction with the modified carbon surface.

PFBA exhibited even weaker adsorption on pristine CAC, with low removal efficiencies and strong dependence on concentration (Fig. 4C). This behavior is consistent with its very short chain length and correspondingly low hydrophobicity, which limits both interaction with the carbon surface and pore-filling contributions.^9,22^ Although both Langmuir and Freundlich models provided reasonable fits, the absence of a clear saturation trend suggests that adsorption occurs across a range of heterogeneous sites without a dominant monolayer mechanism.^20^

EDA functionalization significantly enhanced PFBA adsorption (Fig. 4D), despite a decrease in BET surface area. This further supports the conclusion that adsorption of short-chain PFAS is controlled primarily by surface chemistry rather than physical surface properties.^10^ The introduction of amine groups likely facilitates electrostatic attraction between protonated surface sites and the anionic PFBA headgroup, as well as hydrogen bonding interactions that stabilize adsorption.^11^ However, the isotherm fits were more moderate compared to PFHxA on CTAB–CAC.

### 3.5. Removal efficiency of PFAS on pristine and modified CAC

The removal efficiencies of the four PFAS compounds on pristine CAC and surface-modified carbons are summarized in Fig. 5 as a function of the initial PFAS concentration (1–16 µg L^−1^). Clear differences in adsorption performance were observed depending on PFAS molecular structure and surface modification of the sorbent. For reference, PFOSA (perfluorooctanesulfonamide, C8, MW ≈ 499 g mol^−1^) and PFNA (perfluorononanoic acid, C9, MW ≈ 464 g mol^−1^) are long-chain PFAS, while PFHxA (perfluorohexanoic acid, C6, MW ≈ 314 g mol^−1^) and PFBA (perfluorobutanoic acid, C4, MW ≈ 214 g mol^−1^) are short-chain PFAS (see Introduction for the chain-length definitions used); among these four compounds, PFOSA is generally the most hydrophobic (highest log *K*_ow_) and PFBA the least hydrophobic (lowest log *K*_ow_).

**Fig. 5:**
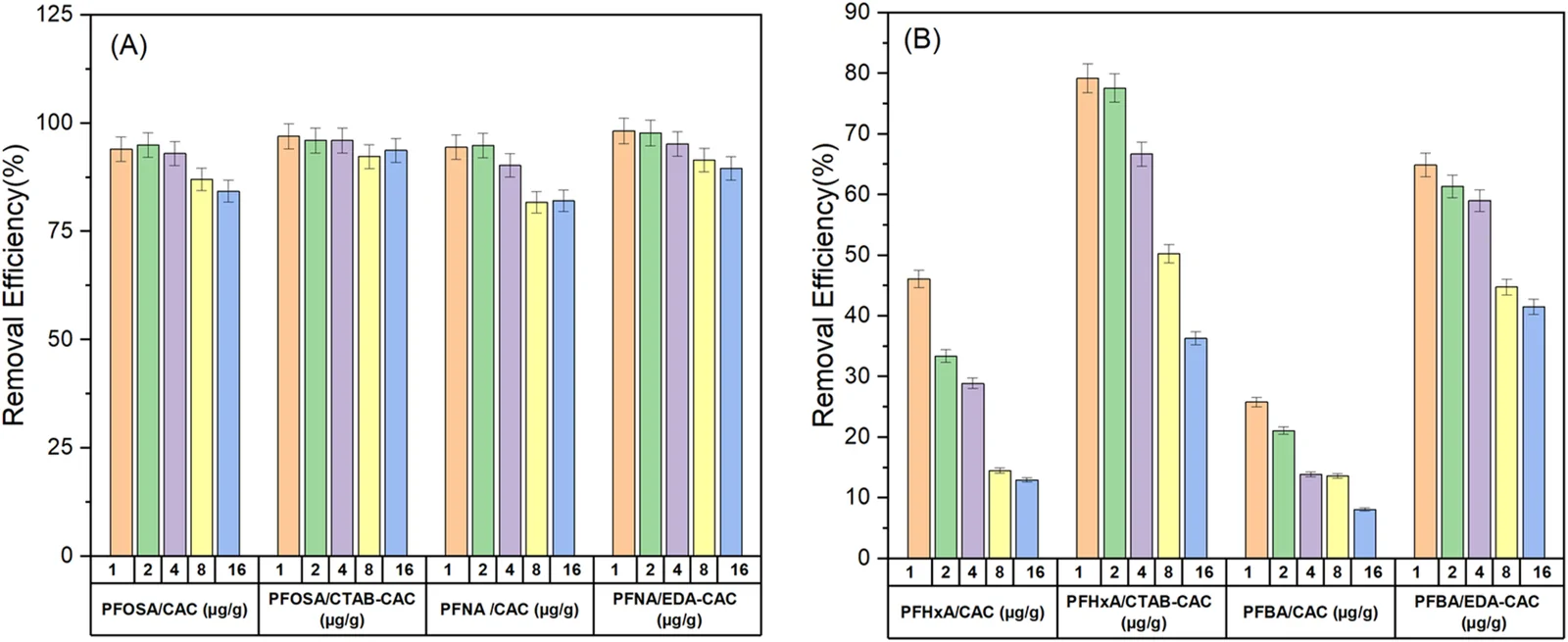
Removal efficiency (%) of four PFAS by coconut activated carbon (CAC) and surface-modified CAC as a function of initial concentration (1, 2, 4, 8, and 16 µg L^−1^) at a sorbent dose of 50 mg L^−1^. (A) Comparison of PFOSA and PFNA adsorption on pristine CAC and their respective modified carbons (CTAB–CAC for PFOSA and EDA–CAC for PFNA). (B) Removal performance of short-chain PFAS (PFHxA and PFBA) on pristine CAC and modified CAC (CTAB–CAC for PFHxA and EDA–CAC for PFBA).

#### 3.5.1. Long-chain PFAS removal

As shown in Fig. 5A, the long-chain PFAS compounds PFOSA and PFNA exhibited consistently high removal efficiencies on both pristine and modified CAC. PFOSA removal on pristine CAC remained above approximately 85–90% across the tested concentration range, demonstrating the strong affinity of long-chain PFAS for activated carbon materials. This behavior is consistent with the strong hydrophobic interactions and van der Waals forces between the extended perfluorinated carbon chain and the aromatic carbon framework of activated carbon.^8,22^ Surface modification had only a modest influence on PFOSA removal. CTAB–CAC showed slightly improved removal efficiencies at lower concentrations compared with pristine CAC, suggesting that surfactant functionalization enhances hydrophobic partitioning between the PFOSA fluorinated chain and the CTAB alkyl groups. However, because pristine CAC already provides strong adsorption sites for long-chain PFAS, the improvement after modification is relatively limited. Similarly, PFNA exhibited high removal efficiencies on both pristine CAC and EDA-modified CAC. Although pristine CAC achieved removals near 90% at low concentrations, EDA–CAC maintained consistently higher removal efficiencies across the concentration range. This improvement can be attributed to the presence of amine functional groups introduced during EDA modification, which create positively charged surface sites capable of electrostatic attraction with the anionic PFNA carboxylate headgroup.^22^ These interactions supplement the inherent hydrophobic adsorption mechanism and enhance overall PFNA uptake.

#### 3.5.2. Short-chain PFAS removal

In contrast, the removal efficiencies of the short-chain PFAS compounds PFHxA and PFBA were significantly lower on pristine CAC and showed a stronger dependence on surface modification (Fig. 5B). For PFHxA, pristine CAC achieved moderate removal efficiencies that decreased substantially with increasing initial concentration. This behavior reflects the weaker hydrophobic interactions associated with shorter perfluorinated chains, which reduce the driving force for adsorption on carbon surfaces.^28^ CTAB modification dramatically improved PFHxA removal efficiency, particularly at lower concentrations. The presence of hydrophobic alkyl chains and positively charged quaternary ammonium groups on the CTAB-functionalized surface likely enhances adsorption through a combination of hydrophobic partitioning and electrostatic attraction.^12,34^ These additional interactions compensate for the inherently weaker hydrophobicity of short-chain PFAS and significantly improve sorption performance.

A similar trend was observed for PFBA, which exhibited the lowest removal efficiencies among the studied compounds due to its short perfluorinated chain length. Pristine CAC removed only a small fraction of PFBA, particularly at higher concentrations. However, EDA modification substantially enhanced PFBA removal efficiency across the tested concentration range. The improvement suggests that electrostatic interactions between protonated amine groups and the anionic PFBA headgroup play an important role in promoting adsorption of short-chain PFAS species that otherwise exhibit limited affinity for carbon surfaces.^9,20,26^ For pristine CAC, removal efficiency followed the general order PFOSA > PFNA > PFHxA > PFBA; the same order was not observed identically across the modified sorbents, since EDA–CAC and CTAB–CAC were each tested against only two of the four compounds (see Section 2.5). This trend reflects the dominant influence of PFAS chain length and hydrophobicity on adsorption behavior.^9,16^ Long-chain PFAS possess stronger hydrophobic character and exhibit greater affinity for carbon surfaces through hydrophobic partitioning and pore-filling mechanisms. In contrast, short-chain PFAS are more water soluble and interact less strongly with carbonaceous adsorbents, resulting in lower adsorption efficiencies.^10,28^

### 3.6. Mechanistic pathways of PFAS adsorption

The mechanistic pathways responsible for PFAS adsorption on pristine and surface-modified coconut activated carbon are illustrated in Fig. 6. The adsorption behavior observed experimentally can be rationalized by considering the interplay between PFAS molecular structure and the surface chemistry of the carbon matrix. Adsorption on coconut-derived activated carbon occurs through a combination of pore confinement, hydrophobic partitioning, electrostatic interactions, and hydrogen bonding, with the relative contribution of each mechanism depending on both the functional groups present on the sorbent surface and the physicochemical properties of the PFAS molecules. For pristine CAC (Fig. 6A), adsorption is primarily governed by hydrophobic interactions between the perfluoroalkyl chains of PFAS and the aromatic carbon domains of the activated carbon structure. The graphitic framework of activated carbon provides a nonpolar environment that promotes partitioning of the fluorinated tail from the aqueous phase into the carbon pore network. These interactions are further enhanced by the highly microporous structure of coconut-derived activated carbon, where confinement within pores increases van der Waals interactions and stabilizes adsorbed molecules through pore-filling effects.^33,38^ Consequently, PFAS molecules with longer perfluoroalkyl chains, such as PFNA, exhibit stronger adsorption affinity because their extended hydrophobic chains provide greater intermolecular contact with the carbon surface.^5,42^ In contrast, short-chain PFAS exhibit weaker adsorption due to their reduced hydrophobic character and higher aqueous solubility.^25^ Oxygen-containing functional groups present on the carbon surface (*e.g.*, hydroxyl, carbonyl, and carboxyl groups) may also participate in weak hydrogen bonding with PFAS headgroups, although these interactions are secondary relative to hydrophobic partitioning.

**Fig. 6:**
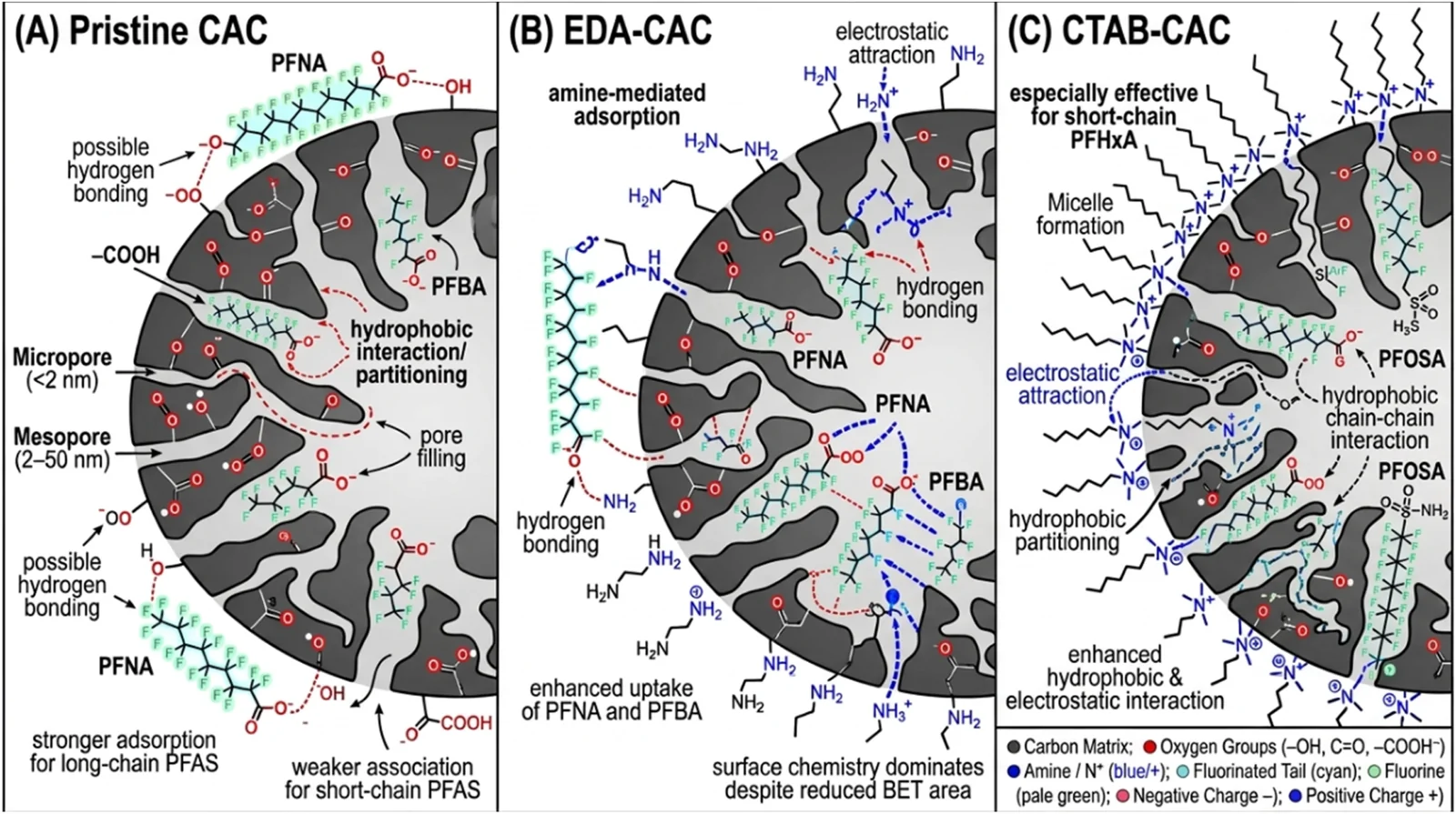
Proposed mechanistic pathways for PFAS adsorption on pristine and surface-modified CAC. (A) Pristine CAC: adsorption is dominated by pore filling within micro- and mesopores and hydrophobic partitioning between the fluorinated PFAS chains and the aromatic carbon framework; secondary interactions may include hydrogen bonding with oxygen-containing surface groups (–OH, –C=O, –COOH). (B) EDA–CAC: surface functionalization with ethylenediamine introduces amine groups that promote adsorption through electrostatic attraction between protonated amine sites and anionic PFAS headgroups, together with hydrogen bonding interactions, enhancing uptake of PFNA and PFBA despite reduced BET surface area. (C) CTAB–CAC: modification with cetyltrimethylammonium bromide introduces hydrophobic alkyl domains and positively charged quaternary ammonium groups that enhance PFAS adsorption through hydrophobic chain–chain interactions and electrostatic attraction, particularly improving adsorption of short-chain PFAS such as PFHxA.

Surface functionalization with ethylenediamine significantly modifies the adsorption environment by introducing nitrogen-containing functional groups onto the carbon surface (Fig. 6B). Under the experimental pH conditions, a portion of these amine groups becomes protonated, forming positively charged ammonium sites capable of interacting electrostatically with anionic PFAS headgroups such as the carboxylate groups present in PFNA and PFBA. These coulombic interactions enhance adsorption affinity by stabilizing PFAS molecules at the carbon–solution interface. In addition, neutral amine groups can participate in hydrogen bonding with PFAS functional groups, further strengthening adsorption complexes. These additional chemical interactions compensate for the slight reduction in BET surface area observed after EDA modification and demonstrate that adsorption behavior is governed more strongly by surface chemistry than by surface area alone.

Modification with CTAB introduces a distinct interfacial environment characterized by long hydrophobic alkyl chains and permanently charged quaternary ammonium groups (Fig. 6C). The surfactant molecules adsorbed onto the carbon surface create hydrophobic domains that enhance PFAS adsorption through hydrophobic chain–chain interactions between the CTAB alkyl groups and the perfluoroalkyl tails of PFAS molecules. At the same time, the positively charged quaternary ammonium groups provide electrostatic interaction sites capable of attracting negatively charged PFAS headgroups. The coexistence of hydrophobic partitioning and electrostatic attraction produces a synergistic adsorption mechanism that increases PFAS affinity for the modified carbon surface. This effect is particularly important for short-chain PFAS, which typically exhibit weak adsorption on pristine activated carbon due to their lower hydrophobicity. The CTAB-modified surface effectively reduces the energetic barrier for adsorption by providing both hydrophobic domains and electrostatic binding sites.

## 4. Conclusion

This study demonstrates that PFAS adsorption onto coconut-activated carbon is governed by the combined effects of chain length, surface chemistry, and the operational concentration range. Across all systems, adsorption affinity followed the order PFOSA > PFNA > PFHxA > PFBA, confirming that increasing perfluorinated chain length strengthens hydrophobic partitioning and pore-filling within the carbon matrix. Pristine CAC exhibited strong and consistent performance for long-chain PFAS, particularly PFOSA and PFNA, where adsorption was dominated by high-affinity interactions and heterogeneous surface sites under sub-saturation conditions. In contrast, short-chain PFAS displayed weaker uptake and greater dependence on surface modification to achieve meaningful removal. Surface functionalization significantly altered adsorption behavior without increasing surface area. CTAB modification substantially enhanced PFHxA adsorption by introducing hydrophobic interfacial domains that improved partitioning of short-chain PFAS, while EDA functionalization increased PFNA affinity through amine-mediated interactions such as electrostatic attraction and hydrogen bonding, despite reduced BET surface area. Isotherm analysis indicated that adsorption across all systems occurred below true monolayer saturation, with Freundlich behavior frequently providing the best description of heterogeneous surface interactions. These findings highlight the importance of tailoring sorbent surface chemistry to PFAS structure and treatment objectives. Surface-engineered carbons are particularly advantageous for low-concentration drinking-water polishing, whereas pristine activated carbon remains highly effective and economical for long-chain PFAS under higher contaminant loading conditions.

This study is subject to several limitations that should guide future work. First, PFSAs (*e.g.*, PFOS, PFHxS, PFBS), which are generally more toxic and bioaccumulative than the PFCAs examined here, were outside the present scope; their inclusion, alongside mixed PFCA/PFSA panels under competitive conditions, is a priority for follow-up studies, particularly given that competitive co-adsorption has been shown to suppress individual PFAS removal in mixed systems.^44^ Second, each modified sorbent was tested against only two of the four PFAS compounds; testing both EDA–CAC and CTAB–CAC against all four compounds would allow a fuller cross-comparison. Third, desorption and regeneration of the spent sorbents were not evaluated in this study. Establishing regeneration feasibility, for example following the closed-loop CTAB-activated-carbon approach demonstrated by Shaikh *et al.*,^34^ is important for the economic and environmental viability of these materials. Finally, isotherm experiments did not reach true monolayer saturation for several systems; future work should extend the concentration range to confirm maximum adsorption capacities, evaluate performance under continuous-flow (fixed-bed/column) conditions relevant to full-scale treatment, and test multi-PFAS mixtures in real water matrices rather than single-compound systems in deionized water.

## Conflicts of interest

There are no conflicts to declare.

## Supplementary Material

RA-OLF-D6RA07028K-s001

## Data Availability

Data will be available upon reasonable request. Supplementary information (SI) is available. See DOI: https://doi.org/10.1039/d6ra07028k.
