# Supplementary material for: Surface functionalization of coconut-shell-derived activated carbon with amine and surfactant groups for selective short-chain PFAS removal
Source: RSC Adv. 2026 Oct 5;16(46):59902–16. doi: 10.1039/d6ra07028k (PMC13635900; doi:10.1039/d6ra07028k)
Supplement: Supplementary file 1 [file RA-016-D6RA07028K-s001.pdf]

Supplementary Information (SI) for RSC Advances.
This journal is © The Royal Society of Chemistry 2026
Supplementary Information (SI) for RSC Advances.
This journal is © The Royal Society of Chemistry

# Surface Functionalization of Coconut-Shell-Derived Activated Carbon with Amine and Surfactant Groups for Selective Short-Chain PFAS Removal

Yasaman Mohammadi[3], Hamidreza Sharifan[1,2*]

[1]Department of Chemistry and Biochemistry, Florida Atlantic University, Boca Raton, FL 33431, USA

[2] School of Environmental, Coastal, and Ocean Sustainability (ECOS), Florida Atlantic University, Boca Raton, FL 33431, USA

[3]Department of Chemistry and Biochemistry, University of Texas at El Paso, Texas, US.

*Corresponding author. Tel: +1-757-287-6370 E-mail: hsharifan@fau.edu

## Supporting Information

The Supporting Information (SI) provides additional details relevant to PFAS identification and adsorption modeling. Table S1 summarizes the target PFAS investigated in this study, including compound names, Formula, m/z (conformation ion), pKa, and water solubility for (perfluorobutanoic acid, PFBA; perfluorohexanoic acid, PFHxA; perfluorononanoic acid, PFNA; and perfluorooctanesulfonamide, PFOSA). Table S2 reports the fitted Langmuir and Freundlich isotherm parameters for PFOSA, PFHxA, PFNA, and PFBA adsorption on Coconut activated carbon (CAC) and surface-modified samples (CTAB-CAC and EDA-CAC), including $q_{max}$, $K_L$, and $R^2$(Langmuir) as well as $K_F$, $n$, and $R^2$(Freundlich).

**Table S1.** Per- and polyfluoroalkyl substances (PFAS) investigated in this study, including compound names, abbreviations, and chemical structures.

| **Compound name** | **Formula** | ***m/z* (confirmation ion)** | **pKa** | **Water solubility (mg/L, 25 °C)** |
|---|---|---|---|---|
| Perfluorooctanesulfonamide (PFOSA) | $C_8H_2F_{17}NO_2S$ | 498.96 | 6.5[1, 2] | 0.37[1, 3] |
| Perfluorohexanoic acid (PFHxA) | $C_6HF_{11}O_2$ | 312.97 | 0.22[1, 3] | 43300[1, 3] |
| Perfluorononanoic acid (PFNA) | $C_9HF_{17}O_2$ | 462.97 | -0.21[2,] | 9500[1, 3] |

| Compound name | Formula | *m/z* (confirmation ion) | pKa | Water solubility (mg/L, 25 °C) |
|---|---|---|---|---|
| | | | [3] | |
| Perfluorobutanoic acid (PFBA) | $C_4HF_7O_2$ | 212.98 | 0.08[2, 3] | 214000[1, 3] |

**Table S2.** Langmuir and Freundlich isotherm parameters for adsorption of PFOSA, PFHxA, PFNA, and PFBA on CAC, CTAB-CAC and EDA-CAC.

| Char material | Langmuir | | | Freundlich | | |
|---|---|---|---|---|---|---|
| | $q_{max}$ | $K_L$ | $R^2$ | $K_F$ | n | $R^2$ |
| PFOSA/CAC | 350 | 1 | 0.95 | 150.31 | 1.62 | 0.98 |
| PFOSA/CTAB-CAC | 350 | 2.5 | 0.87 | 287.68 | 1.15 | 0.97 |
| PFHxA/CAC | 44.44 | 0.33 | 0.85 | 11 | 2 | 0.89 |
| PFHxA/CTAB-CAC | 130.70 | 0.55 | 0.99 | 44.84 | 2.4 | 0.99 |
| PFNA/CAC | 350 | 1 | 0.93 | 121.98 | 1.46 | 0.96 |
| PFNA/EDA-CAC | 350 | 1.5 | 0.97 | 205.89 | 1.61 | 0.99 |
| PFBA/CAC | 50 | 0.1 | 0.94 | 6.91 | 1.91 | 0.96 |
| PFBA/EDA-CAC | 55.84 | 0.79 | 0.82 | 23.63 | 2.7 | 0.85 |

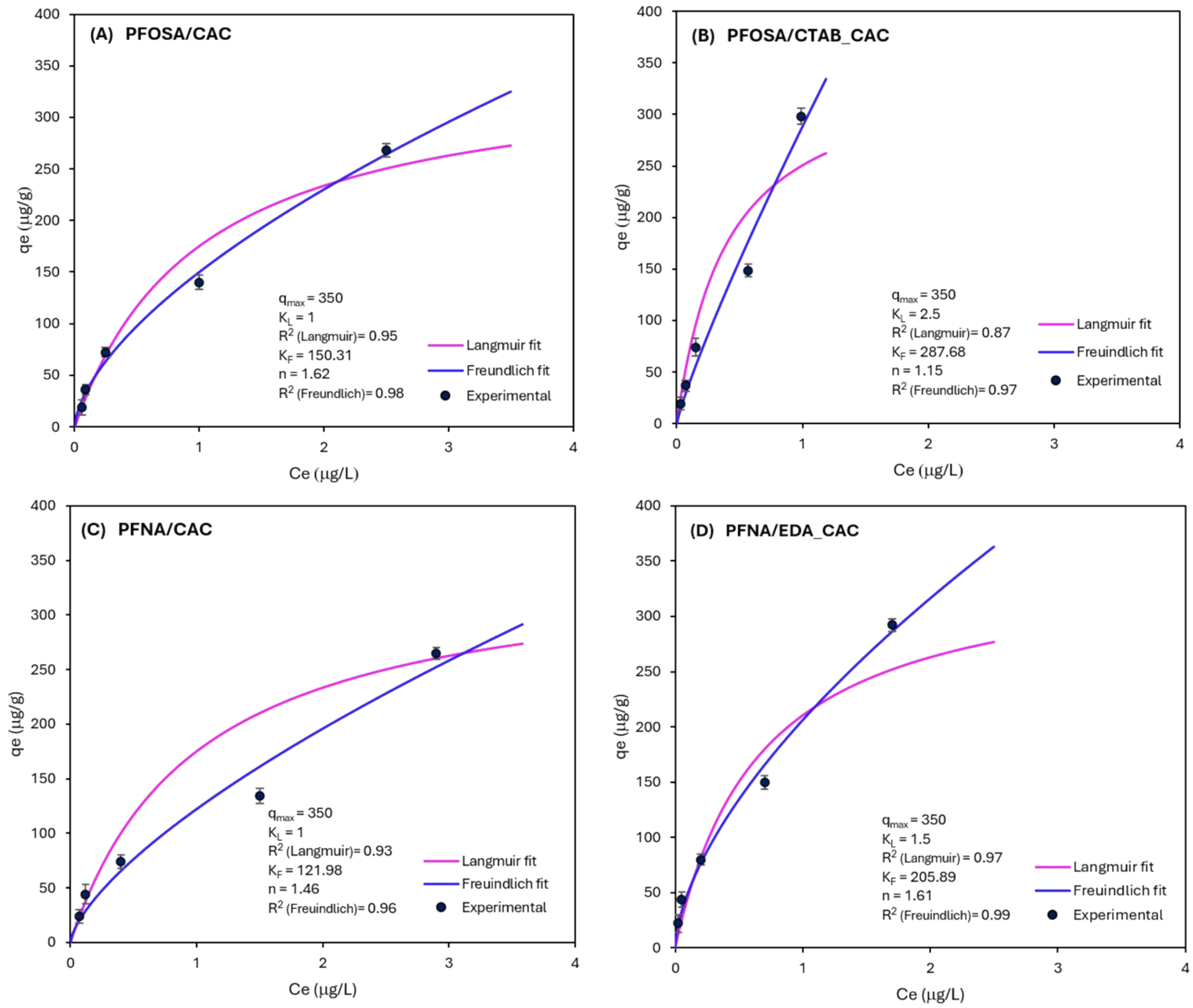


**Figure S1.** Langmuir and Freundlich isotherm model fits for PFAS adsorption on pristine coconut activated carbon (CAC) and surface-functionalized CAC: (A) PFOSA on CAC, (B) PFOSA on CTAB-CAC, (C) PFNA on CAC, and (D) PFNA on EDA-CAC. Data points represent mean ± SD (n = 3).

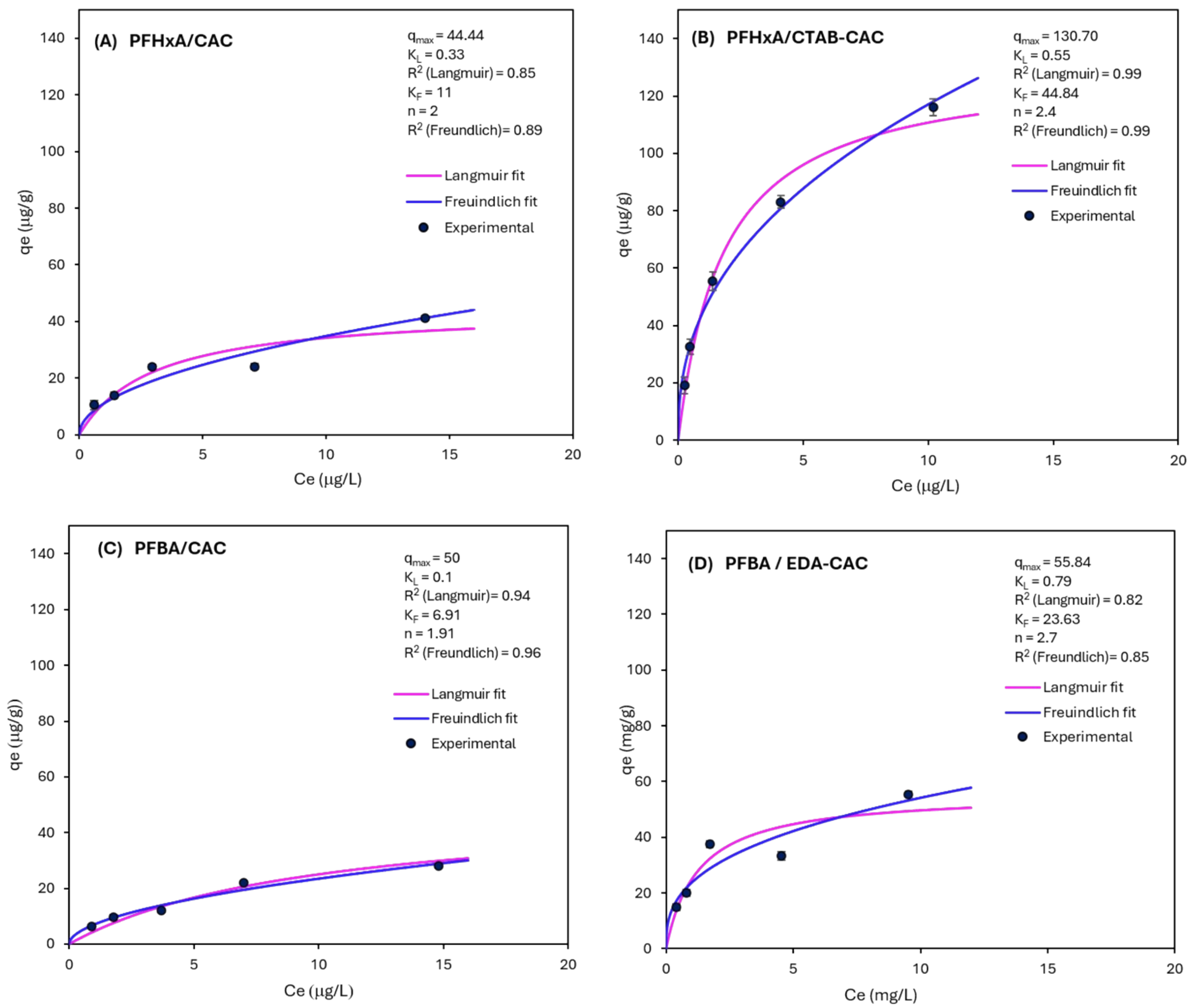


**Figure S2.** Langmuir and Freundlich isotherm model fits for PFAS adsorption on pristine coconut activated carbon (CAC) and surface-functionalized CAC: (A) PFHxA on CAC, (B) PFHxA on CTAB-CAC, (C) PFBA on CAC, and (D) PFBA on EDA-CAC. Error bars represent the standard deviation (SD) of replicate LC–MS/MS measurements.

PFBA

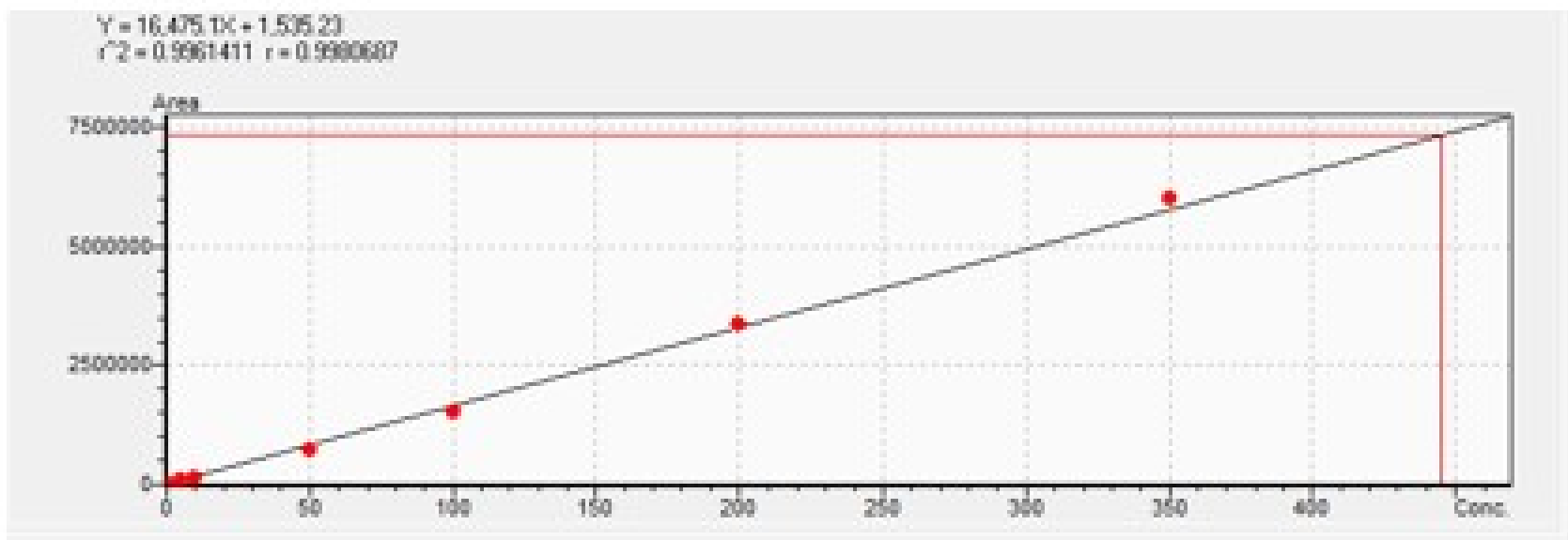


PFHxA

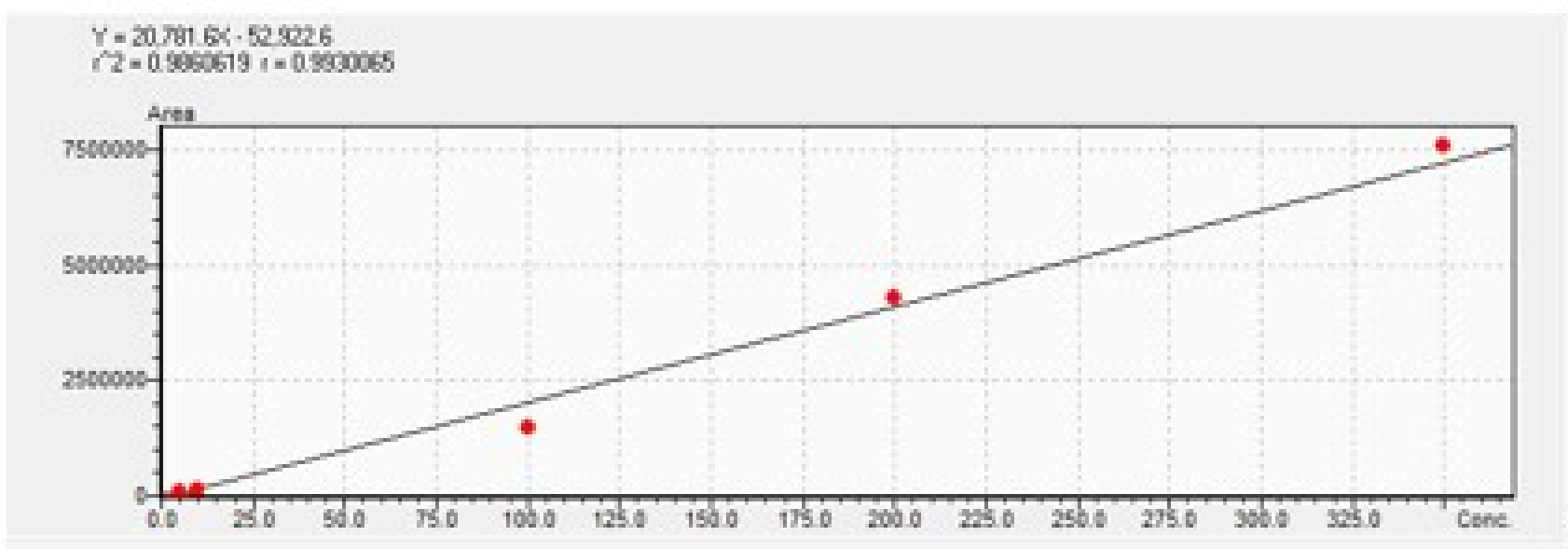


PFNA

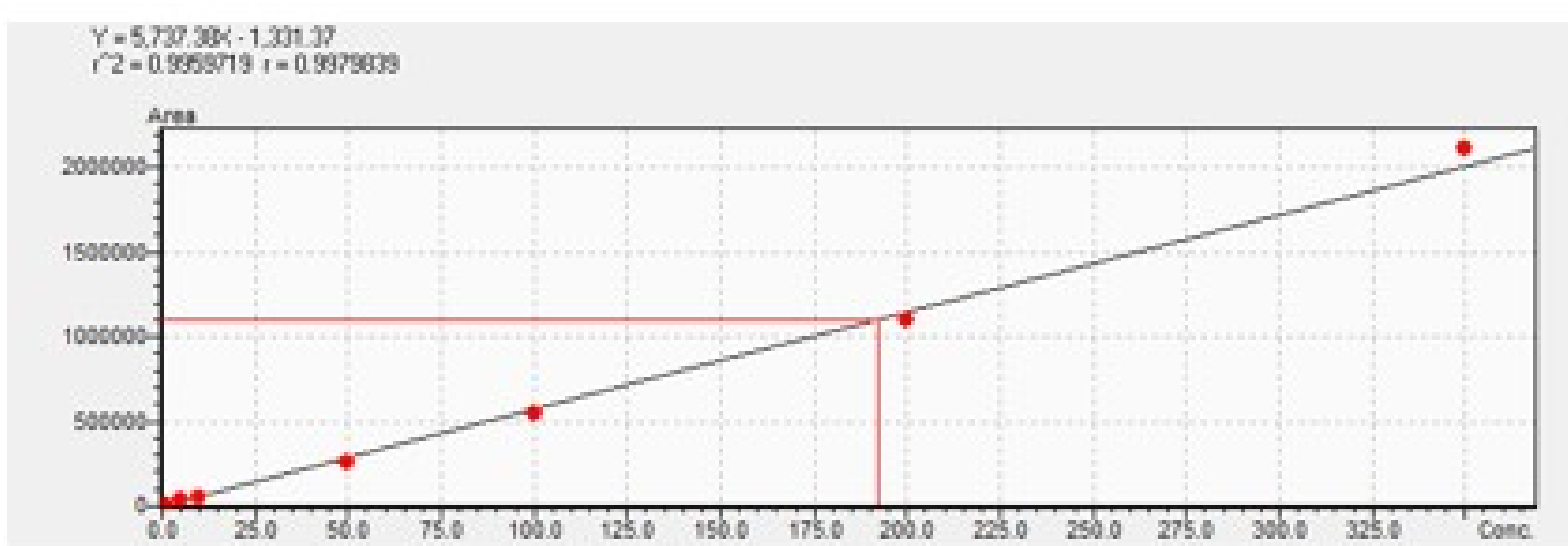


PFOSA

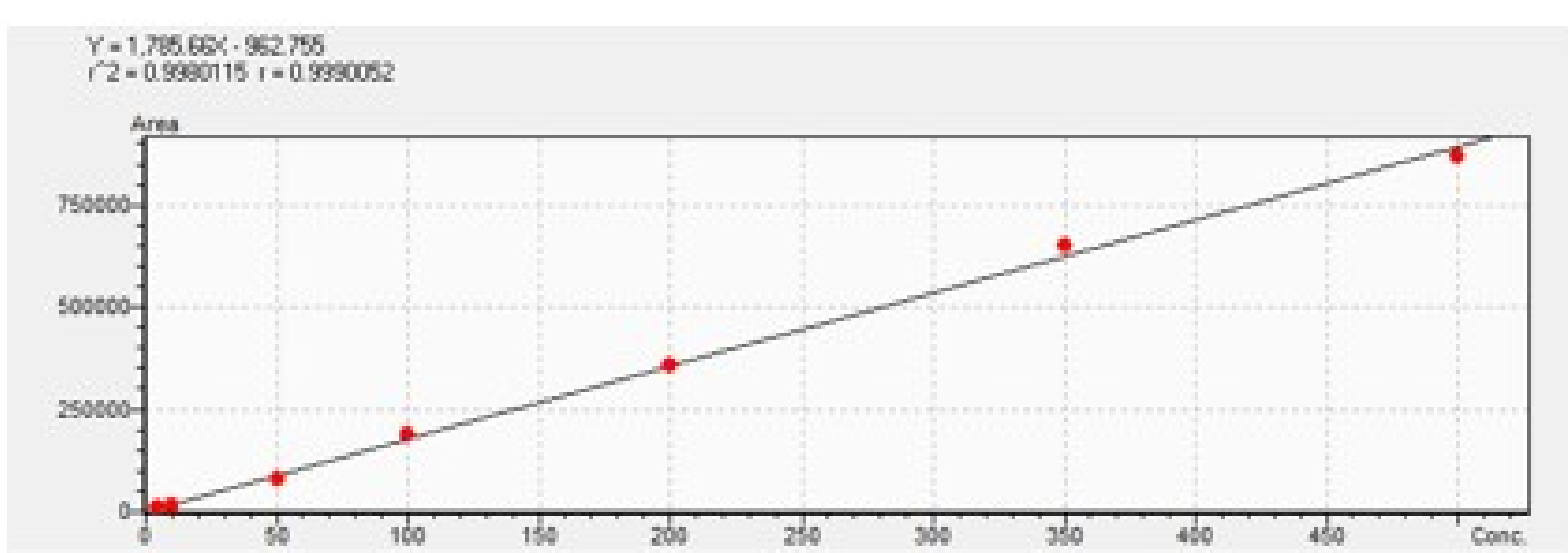


**Figure S3.** Representative LC–MS/MS calibration curves for the target PFAS analytes, shown in the order labeled above: PFBA, PFHxA, PFNA, and PFOSA. For each analyte, chromatographic

peak area is plotted against analyte concentration; the linear regression equation and correlation coefficients ($r^2$ and r) are displayed on each panel.

## References

(1) Wang, Z.; Buser, A. M.; Cousins, I. T.; Demattio, S.; Drost, W.; Johansson, O.; Ohno, K.; Patlewicz, G.; Richard, A. M.; Walker, G. W. A new OECD definition for per-and polyfluoroalkyl substances. *Environmental science & technology* **2021**, *55* (23), 15575-15578.
(2) Buck, R. C.; Franklin, J.; Berger, U.; Conder, J. M.; Cousins, I. T.; De Voogt, P.; Jensen, A. A.; Kannan, K.; Mabury, S. A.; Van Leeuwen, S. P. Perfluoroalkyl and polyfluoroalkyl substances in the environment: terminology, classification, and origins. *Integrated environmental assessment and management* **2011**, *7* (4), 513-541.
(3) Goss, K.-U. The p K a values of PFOA and other highly fluorinated carboxylic acids. *Environmental science & technology* **2008**, *42* (2), 456-458.
